# Intraputaminal Gene Delivery in Two Patients with Aromatic L‐Amino Acid Decarboxylase Deficiency

**DOI:** 10.1002/mdc3.13685

**Published:** 2023-02-24

**Authors:** Marie‐Céline François‐Heude, Gaetan Poulen, Emmanuel Flamand Roze, Marie‐Ange Nguyen Morel, Domitille Gras, Isabelle Roch‐Toreilles, Adeline Quintard, Gaelle Baroux, Pierre Meyer, Philippe Coubes, Christophe Milesi, Gilles Cambonie, Julien Baleine, Chrystelle Sola, Bénédicte Delye, Evgenia Dimopoulou, Stéphanie Sanchez, Mathieu Gasnier, Souad Touati, Alberto Zamora, Daniel Pontal, Nicolas Leboucq, Virginie Kouyoumdjian, Adrien Lebasnier, Sylvia Sanquer, Denis Mariano‐Goulart, Thomas Roujeau, Agathe Roubertie

**Affiliations:** ^1^ CHU Montpellier, Département de Neuropédiatrie Univ Montpellier Montpellier France; ^2^ Département de Neurochirurgie CHU Montpellier Montpellier France; ^3^ Assistance Publique ‐ Hôpitaux de Paris CHU Pitié‐Salpêtrière DMU Neurosciences et Sorbonne Université, INSERM, CNRS, Institut du Cerveau et de la Moelle Paris France; ^4^ Service de Neurologie Pédiatrique Hôpital Couple Mère Enfant, CHU Grenoble Alpes La Tronche France; ^5^ U1141 Neurodiderot, Équipe 5 inDev, Inserm, CEA, UP, UNIACT, Neurospin, Joliot, DRF, CEA‐Saclay Paris France; ^6^ Pharmacie, CHU Montpellier Montpellier France; ^7^ PhyMedExp, CNRS, INSERM, Université de Montpellier Montpellier France; ^8^ Département de Réanimation Pédiatrique CHU Montpellier Montpellier France; ^9^ Département d'Anesthésie‐Réanimation CHU Montpellier; Institute of Functional Genomics (IGF), Université de Montpellier, CNRS, INSERM Montpellier France; ^10^ Département d'Anesthésie‐Réanimation CHU Gui de Chauliac Montpellier France; ^11^ Département de MPR Institut Saint Pierre Palavas France; ^12^ Département de Neuroradiologie CHU Montpellier Montpellier France; ^13^ Département de Médecine Nucléaire CHU Montpellier Montpellier France; ^14^ Département de Biologie Métabolique APHP Paris France; ^15^ INM, Univ Montpellier, INSERM U 1298 Montpellier France

**Keywords:** AADC deficiency, elodecagene exuparvovec, gene therapy, oculogyric crisis

## Abstract

**Background:**

Aromatic l‐amino acid decarboxylase deficiency (AADCD) is a rare, early‐onset, dyskinetic encephalopathy mostly reflecting a defective synthesis of brain dopamine and serotonin. Intracerebral gene delivery (GD) provided a significant improvement among AADCD patients (mean age, ≤6 years).

**Objective:**

We describe the clinical, biological, and imaging evolution of two AADCD patients ages >10 years after GD.

**Methods:**

Eladocagene exuparvovec, a recombinant adeno‐associated virus containing the human complimentary DNA encoding the AADC enzyme, was administered into bilateral putamen by stereotactic surgery.

**Results:**

Eighteen months after GD, patients showed improvement in motor, cognitive and behavioral function, and in quality of life. Cerebral l‐6‐[^18^F] fluoro‐3, 4‐dihydroxyphenylalanine uptake was increased at 1 month, persisting at 1 year compared to baseline.

**Conclusion:**

Two patients with a severe form of AADCD had an objective motor and non‐motor benefit from eladocagene exuparvovec injection even when treated after the age of 10 years, as in the seminal study.

Aromatic l‐amino acid decarboxylase deficiency (AADC) deficiency (AADCD; OMIM 608643) is a rare autosomal recessive disease because of pathogenic variants of the dopa decarboxylase (*DDC* gene); subsequent monoamine defect leads to early‐onset disorder characterized by hypotonia, movement disorders, dysautonomic features, and behavioral disturbances.^1^, ^2^, ^3^, ^4^, ^5^ Medications are disappointing, and 80% of the patients suffer from severe motor and cognitive impairment and risk of premature death.^3^, ^6^ Gene delivery (GD) for AADCD provides the defective gene by injecting a recombinant adeno‐associated virus containing the human complimentary DNA encoding the *DDC* gene, such as eladocagene exuparvovec, directly into the brain to increase striatal synthesis of dopamine.^7^ Thirty‐nine young patients with AADCD (mean age, ≤6 years old) have benefited from intraputaminal GD,^8^, ^9^, ^10^, ^11^ and seven from midbrain GD,^12^ with significant improvement in motor and non‐motor functions. Here, we report the 18‐month follow‐up of the first two patients >10 years old treated in Europe by intraputaminal eladocagene exuparvovec delivery.

## Materials and Methods

### Selection of the Patients

Patients were referred for GD by their local pediatric neurologist and were selected as candidates for eladocagene exuparvovec injection based on their symptomatic presentation, lack of improvement on traditional standard of care, and clinical suitability for GD by a multidisciplinary multicentric group of experts. Eladocagene exuparvovec was available under a temporary authorization for compassionate use, according to the article L.5121‐12 of the Public Health Code, in Montpellier University Hospital. Baseline and post‐GD assessment schedule of the patients and neurosurgical procedure according to the fabricant recommendations have been validated by the French Drug Agency as daily care including expenses support.

### Surgical Procedure

Each patient received a total of 1.8 × 10^11^ viral genomes delivered as four intraputaminal injections of 80 μL conducted at a rate of 3 μL/min. Protocols for surgery, pharmacy, storage, and shipment of eladocagene exuparvovec have been previously described.^7^, ^13^

### Clinical and Paraclinical Assessment

Clinical data were collected in the medical file and at each follow‐up visit, specifically to motor function (Gross Motor Function Measure–88 Item, Manual Ability Classification System), cognitive function (Vineland Adaptive Behavior Scales), sleep and mood disturbances, irritability, comfort, wellbeing, and ease of caregiving according to Caregiver Priorities and Child Health Index of Life with Disabilities (CPCHILD) questionnaire, gastrointestinal and feeding difficulties, weight, respiratory infections, pharmacological changes, dyskinesia, and oculogyric crisis (Pearson's score).^12^ Brain magnetic resonance imaging, l‐6‐[^18^F] fluoro‐3, 4‐dihydroxyphenylalanine (^18^F‐DOPA) positron emission tomography (PET) were performed before GD and 1 month and 1 year after GD. Cerebrospinal fluid analysis (cell count, biochemical analysis, and neurotransmitters' profile) was performed before GD and 18 months after GD. Anti–adeno‐associated virus type 2 titers were monitored at baseline and at 3, 6, 12, and 18 months after GD.

## Results

Patient 1 and 2 benefited from GD (1.8 × 10^11^ viral genomes delivered as four intraputaminal injections) at ages 10.7 years and 11.4 years, respectively. Past medical history is summarized in Table S1. Before GD, patients had no head control and limited voluntary movement despite treatment strategy conducted according to the guidelines (1, 4).^3^ Longitudinal assessment data are summarized in Table 1.

**TABLE 1:** Clinical and biological parameters at baseline and 3, 6, 12, and 18 months after gene delivery

	Patient 1					Patient 2				
	Baseline	M3	M6	M12	M18	Baseline	M3	M6	M12	M18
Motor signs										
Head control	−	−	−	+ (10 m)	+	−	−	+	+	+
Hand function	−	−	Can reach	Can reach	Can reach	−	−	Can grab on contact	Can grab (8 m)	Can grab
Spontaneous movements	Almost none	+ (LL)	+ (limbs)	++	+++	−	+/− (head)	+ (head, limbs)	++ (left>right)	+++ (left>right)
GMFM‐88 (global %/target %)	1,96	3,41	6,43	5,31	9,06	2,35	3,74	5,64	6,31	6.69
MACS (level)	V	V	V	V	V	V	V	V	V	V
Movement disorders										
Oculogyris crises (Pearson's score)	16 twice a week involving the eyes and face lasting 4 hours	12	6	5,6	0, 2 1–2 per month subtle involving the eyes lasting 10 minutes	4/y (0,15)	0	0	0	0
Dystonia	++ Distal	+ (limbs)	+/−	+/− (LL)	+/− (LL)	−	+ (LL/face)	+/− (LL/face)	+/− (oral)	+/− (mouth opening)
Dyskinesia	3	36	48	4 (significant improvement from month 8)	1	0	24	9 (significant improvement from month 5)	0	0
Comportement and cognitive assessment										
Sleep disturbances	3	0 (1 m)	0	0	0	3	0 (1 m)	0	0	0
Mood disturbances (crying)	3	0 (1 m)	0	0	0	3	0 (1 m)	0	0	0
CPCHILD section 3 (%)	79	100 (more comfortable)	90	97	92	47	95 (more comfortable)	100	96	92
Communication	Eye contact, grunt	Vocalize, smile	3 Sounds, laughs	9 Sounds, babbling, best social interactions	Bisyllabic words, « papa »	Eye contact, eye communication, 1 sound	Better eye communication 2 sounds	Recognizes the letters, smile 2 sounds	Smile, more sounds	Vocalize, several sounds, no word, smile
VINELAND communication: Receptive/expressive	4/10	NA	NA	11/13	12/14	12/9	NA	NA	14/12	15/12
VINELAND daily living skills: persona/domestic/community	6/−/−	NA	NA	7/−/1	7/1/−	2/−/−	NA	NA	6/−/2	8/−/3
VINELAND Socialization: interpersonal relationships/play and leisure/coping skills	9/3/2	NA	NA	14/3/2	15/3/2	12/4/2	NA	NA	19/4/4	20/4/4
Digestive and pulmonary function										
Oral/enteral nutrition (%)	100/0	100/0	100/0	100/0	100/0	0/100	20/80	70/30	70/30	100/0
Digestive motility	Normal	Normal	Normal	Normal	Normal	Diarrhea	Diarrhea	Diarrhea	Constipation	−
Weight (kg)	23	25	24.5	27	26.5	27	27.4	28.5	36.2	32.5
Pulmonary infections per years	0	0	0	0	0	6	0	0	0	1
Pharmacological treatments										
No. of specialties	0	1 Tetrabenazine 1.2 mg/kg/day	1 Tetrabenazine 1.2 mg/kg/day	1 Tetrabenazine 0.5 mg/kg/day	0	9 Pyridoxine, rotigotine, amitriptiline, levotonine, melatonine, sodium valproate, clonazepam, azithromycine, esomeprazole	3 Sodium valproate, clonazepam ↓, azithromycine	3 Sodium valproate, clonazepam, azithromycine	4 Sodium valproate, clonazepam ↓, azithromycine macrogol	3 Sodium valproate, clonazepam, macrogol
Pulmonary infections per year	0	0	0	0	0	6	0	0	0	1
CSF Biology										
CSF HVA (normal range: 156–410 nmol/L)	28	NA	NA	NA	15.72	17.02	NA	NA	20.77	31.49
CSF 5‐HIAA (normal range: 63–85 nmol/L)	<20*				<1*	2.88*			<1¨	1,71*
CSF 3OMD (normal range: 3–54 nmol/L)	462	NA	NA	NA	453	1009	NA	NA	1136	599
Plasma Biology										
Plasma 3‐OMD (normal: < 0.3 μmol/l)	4.24	4.16	5	3.88	4.35	3.48	3.78	3.57	3.7	2.6
AVV2 serology (titer)	<10	<10	<10	<10	<10	184,68	614,8	>10,240	9149	9252

*Note*: The severity of OGC: OGC was calculated according to the Pearson's score^12^: the weekly average of the duration of episodes (h/wk), weighted by severity (grade 1–3: 1 = mild/eye deviation only; 2 = moderate/eye deviation + dystonia or dyskinesia of the face and/or neck; 3 = severe/dystonia or dyskinesia involving the trunk and/or limbs). The severity of sleep and mood disturbances (excessive crying, irritability) were assessed according to a qualitative scale described by Pearson^12^ et al: 3 = severe; 2 = moderate; 1 = slight; 0 = absent. Dyskinesias were assessed by a composite score combining location in 6 body regions (0–6), duration (0–4), and severity (0–4), with a maximum score of 96.* HIAA levels: dramatically reduced values within the same range.

Abbreviations: 3‐OMD, 3‐O methyldopa; 5‐HIAA, 5‐hydroxyindoleacetic acid; AAV2, adeno‐associated virus type 2; CSF, cerebrospinal fluid; GMFM‐88, Gross Motor Function Measure–88 Items; HVA, homovanillic acid; LL, lower limb; OGC, oculogyris crises MACS, Manual Ability Classification System; NA, not available.

**Video 1:** Patient 1, baseline. Limited spontaneous movements, akinesia, lower limb distal dystonia, no head control.

### Clinical Assessment

#### Motor Function

Both patients exhibited improved motor function, especially axial tone and voluntary movements of limbs. Patient 1 could control the head at 10 months, and patient 2 at 6 months. Spontaneous and voluntary movements dramatically improved from 2 months after GD. Patient 1 could reach an object at 6 months, whereas patient 2 could grab from 12 months (2, 3, 5, 6). Dystonia and oculogyric crisis (OGC) (duration, frequency, and severity) diminished progressively from 3 months after GD.

**Video 2:** Patient 1, 18 months after gene delivery. The patient can hold up the head and exhibits spontaneous movement of the upper limbs.

**Video 3:** Patient 1, 18 months after gene delivery. Familial video; the patient can hold up the head when sitting with support.

**Video 4:** Patient 2, baseline. Severe axial hypotonia with no head control, akinesia, limited spontaneous movements with upper limb hypotonia.

**Video 5:** Patient 2, 18 months after gene delivery. The patient can hold up the head when sitting in the wheelchair.

**Video 6:** Patient 2, 18 months after gene delivery. The patient can grab the object with the left arm and bring it to the head.

#### Behavioral and Cognitive Function

At baseline, both patients had mood and sleep disturbances, excessive crying, and could only communicate through eye contact and grunting. From the fourth week after GD, caregivers/families reported improvement of sleep disturbances, irritability, and wakefulness. Joyful behavior, increased alertness, and better communication were also reported for both patients, including patient 1's newly gained ability to laugh from month 6 after GD (Table 1, CP child, section 0). At baseline, patient 1 could only grunt, and patient 2 could not produce any sound. Six months after GD, both patients could emit various sounds and syllables used to call the caregivers; 18 months after GD, patient 1 could emit bisyllabic words. Improvement of cognitive subdomains is highlighted by Vineland subscore (Table 1, Vineland scores).

#### Other Functions

Feeding abilities (including weaning from tube feeding in patient 2) resulted in increased in body weight of both patients; respiratory infections in patient 2 significantly improved (Table 1).

### Adverse Events

No serious adverse events were reported during the surgical procedure or follow‐up. Patient 2, with a known, well‐controlled epilepsy, exhibited a cluster of brief focal seizure during a non‐infectious febrile peak 36 h after GD. Moderate and transient dyskinesia occurred in both patients from 4 weeks following GD; they were controlled with low doses of tetrabenazine in patient 1 and by tapering down dopaminergic drugs in patient 2 (Table 1). Patient 1 exhibited a meningocele at the injection site because of transient cerebrospinal fluid (CSF) leaking with spontaneous improvement.

### Paraclinical Assessment

**FIG. 1:**
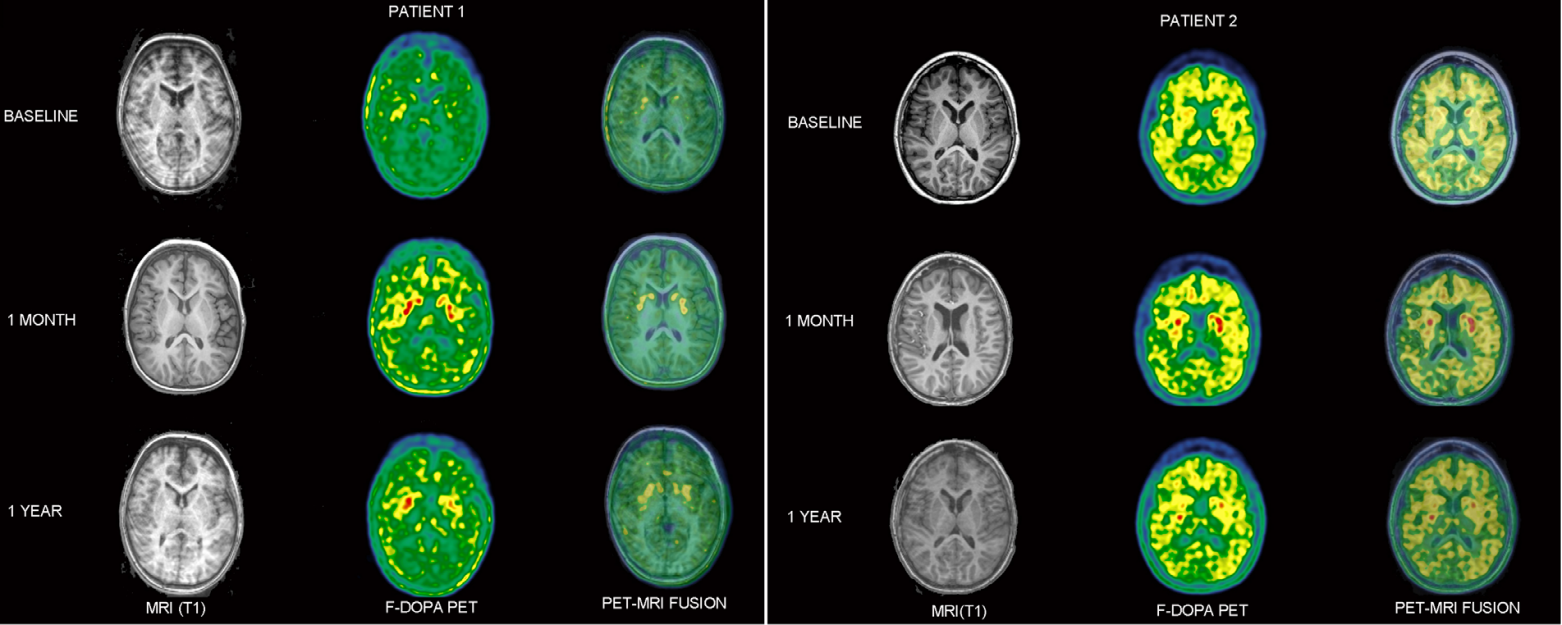
^18^F‐DOPA PET and MRI images at baseline, 1 month, and 1 year after gene delivery. The color palette was centered on the maximum signal observed in the cortical gray matter, to allow visual comparison between time points and patients. ^18^F‐DOPA PET scan visualizes the capacity of the neurons of the putamen to durably take up ^18^F‐DOPA, which implies an efficient decarboxylation of this amino acid. Increased fixation is observed 1 month and 1 year after gene delivery. ^18^F‐DOPA, l‐6‐[^18^F] fluoro‐3, 4‐dihydroxyphenylalanine; PET, positron emission tomography; MRI, magnetic resonance imaging.

Post‐surgery magnetic resonance imaging did not show any feature of bleeding or infection. Both patients showed severe impairment of ^18^F‐DOPA uptake at baseline, with improvement in uptake at 1 month persisting at 1 year compared with baseline, albeit more heterogeneously in patient 2 (Fig. 1 and Table S2).

There was an increased CSF level the dopamine metabolite homovanillic acid (HVA), but not of the serotonin metabolite 5‐hydroxyindoleacetic acid (5‐HIAA) level in patient 2 at last follow‐up compared to baseline. Dopamine levels remained stable in patient 1 (Table 1).

## Discussion

We report the first European compassionate experience of intraputaminal delivery of eladocagene exuparvovec in two children >10 years old with a severe form of AADCD. This well‐tolerated treatment resulted in consistent improvement of motor and non‐motor function. Follow‐up was characterized by early improvement in sleep and mood disturbances, alertness, and communication skills, especially expression abilities. Both patients managed to hold up their head before 8 months. Spontaneous movements were dramatically enriched, allowing the patients to reach/grab objects; increased Gross Motor Function Measure‐88 Item score highlight motor outcome. This progress, as well as resolution of OGC, and ability to feed orally resulted in remarkable improvement of quality of life. Previous studies showed faster and greater progress in young patients, especially regarding gross motor progress and fine motor skills (ability to reach/grab or eat with hands),^7^, ^8^, ^9^, ^11^, ^12^ with an even better functional recovery in patients young than 3;^8^ data concerning patients >10 years old are limited. Indeed, Kojima et al^9^ reported four patients >10 years old with improvement of cognitive and motor functions (head control and sitting with support: all patients before 8 months after GD; stand with support: 3 of 4 patients before 18 months after GD; aided walk: 1 patient; all patients could grip at the last follow‐up) and disappearance of OCG and dystonia following intraputaminal administration of rAAV‐hAADC gene therapy.^9^

Side effects were limited, dyskinesia occurred as reported in other studies;^7^, ^8^, ^9^, ^11^, ^12^ they lasted less than 6 months and were easily controlled.

Because ^18^F‐DOPA is rapidly washed out from putaminal neurons if not metabolized into dopamine, evidence of recovery of a certain level of cerebral AADC activity is provided by recovery of putaminal uptake of ^18^F‐DOPA for both patients 1 year after GD, albeit a partial decrease in uptake compared with uptake 1 month after GD for patient 2. These results are similar to those of Hwu et al,^7^ confirming the importance of ^18^F‐DOPA PET in providing indication of the biological effectiveness of GD.

Restoration of AADC activity is also supported by increased CSF levels of HVA in patient 2. Patient 1's HVA CSF dosage was not increased from baseline to last follow‐up. Although a strong correlation has been reported between the post‐treatment HVA level and motor outcome,^11^ limited changes in HVA levels have been encountered in previously reported patients with significant clinical improvement.^8^, ^12^ Moreover, CSF dopamine metabolites levels after intracerebral *DDC* gene delivery have not been assessed in animal models, therefore, scientific experimental data concerning CSF dopamine metabolites as reliable biomarkers of AADC activity restauration after gene delivery are lacking. The value of CSF biomarkers as a relevant clue for AADC activity restauration and/or prognosis indicator of good motor outcome will need to be further studied with standardized timepoints of assessement.

As in previous series of AADCD patients who received gene delivery, clinical assessment of the two patients was unblinded, and part of evaluation was based on caregivers rating (especially wakefulness, behavior, and sleep);^7^, ^8^, ^9^, ^11^, ^12^ this approach intrinsically includes bias related to the magnitude of expected benefit from the caregivers and clinicians' awareness of the patient's status. Moreover, various and heterogeneous scales or scores have been used among the series,^7^, ^8^, ^9^, ^11^, ^12^ resulting in limitations in the attempts to compare patient's outcome, especially concerning functional improvement, which probably represent one of the most meaningful and significant objective of therapy in such severe disease.

## Conclusion

Robust evidence corroborates the absence of the degenerative process in AADC.^3^, ^14^, ^15^ This basic rationale supports extending gene therapy to patients older than those studied in the pivotal clinical trials. The clinical outcome in our two patients is concordant with previous reports and confirms that intraputaminal DDC gene delivery has a consistent safe profile and can be considered for use in teenagers or older patients suffering from a severe form of AADCD with significant impairments. Unified and standardized monitoring of the patients will aid in better understanding of the biological and clinical effect of this innovative therapeutic procedure.

## Author Roles

(1) Research project/study: A. Conception, B. Organization, C. Execution; (2) Statistical Analysis: A. Design, B. Execution, C. Review and Critique; (3) Manuscript: A. Writing of the First Draft, B. Review and Critique.

S.S.: 1C, 3B.

P.C.: 1C, 3B.

S.S.: 1C, 3B.

I.R.T.: 1C, 3B.

C.M.: 1C, 3B.

P.M.: 1C, 3B.

A.L.: 1C, 3B.

D.G.: 1C, 3B.

G.C.: 1C, 3B.

D.M.G.: 1B, 1C, 3B.

D.P.: 1C, 3B.

J.B.: 1C, 3B.

M.A.N.M.: 1C, 3B.

A.Q.: 1C, 3B.

G.B.: 1C, 3B.

C.S.: 1C, 3B.

B.D.: 1C, 3B.

E.D.: 1C, 3B.

M.G.: 1C, 3B.

S.T.: 1C, 3B.

A.Z.: 1C, 3B.

N.L.: 1C, 3B.

V.K.: 1C, 3B.

E.R.: 1B, 1C, 3B.

M.C.F.H.: 1C, 3A.

G.P.; 1C, 3A.

T.R.: 1A, AB, 1C, 3B.

A.R.: 1A, AB, 1C, 3B.

## Disclosures

**Ethical Compliance Statement**: The authors confirm that the approval of an institutional review board was not required for this work. All legal representative of the patients provided written and oral informed consent according to the Declaration of Helsinki to perform the procedure, collect and share the data including videos. All authors confirm that they have read the Journal's position on issues involved in ethical publication and affirm that this work is consistent with those guidelines.

**Funding Sources and Conflicts of Interest**: The authors have no funding source to declare and no conflicts of interest to declare.

**Financial Disclosures for the Previous 12 Months**: E.R. received honorarium for speech from Orkyn, Aguettant, Elivie and for participating in an advisory board from Merz‐Pharma. He received research support from Merz‐Pharma, Orkyn, Aguettant, Elivie, Ipsen, Everpharma, Fondation Desmarest, AMADYS, Fonds de dotation Patrick Brou de Laurière, Agence Nationale de la Recherche, Société Française de Médecine Esthétique, Dystonia Medical Reasearch Foundation. T.R. received honorarium for advisory board from PTC Therapeutics. A.R. received honorarium for advisory board from PTC Therapeutics. The authors declare that there are no additional disclosures to report.

## Supporting information


**Table S1.** Characteristics and past medical history of patients.
**Table S2.** Quantification of the specific putaminal uptake at baseline, 1 month, and 1 year after GD.
