# Supplementary material for: Intraputaminal Gene Delivery in Two Patients with Aromatic L‐Amino Acid Decarboxylase Deficiency
Source: Mov Disord Clin Pract. 2023 Feb 24;10(5):811–8. doi: 10.1002/mdc3.13685 (PMC10187009; doi:10.1002/mdc3.13685)
Supplement: Supplementary file 1 — Table S1. Characteristics and past medical history of patients. Table S2. Quantification of the specific putaminal uptake at baseline, 1 month, and 1 year after GD. [file MDC3-10-811-s001.docx]

**SUPPLEMENT**

**Innovative Therapy in a Rare Disease: Results of Intraputaminal Gene Delivery in 2 Patients With Aromatic l-Amino Acid Decarboxylase Deficiency**

Marie-Céline François-Heude, MD; Gaetan Poulen, MD; Emmanuel Roze, MD, PhD; Marie-Ange Nguyen Morel, MD; Domitille Gras, MD; Isabelle Roch-Torreilles, PharmaMD; Adeline Quintard, PharmaMD; Gaelle Baroux, PharmaMD; Pierre Meyer, MD, PhD; Philippe Coubes, MD, PhD; Christophe Milesi, MD; Gilles Cambonie, MD, PhD; Julien Baleine, MD; Chrystelle Sola, MD; Bénédicte Delye, MD; Evgenia Dimopoulou, MSc; Stéphanie Sanchez, MSc; Mathieu Gasnier, MSc; Souad Touati, MSc; Alberto Zamora, MSc; Daniel Pontal, MSc; Nicolas Leboucq, MD; Virginie Kouyoumdjian, MD; Adrien Lebasnier, MD; Sylvia Sanquer, MD; Denis Mariano-Goulart, MD, PhD; Thomas Roujeau, MD, PhD; Agathe Roubertie, MD, PhD

- **Supplementary Table 1.** Characteristics and Past Medical History of Patients
- **Supplementary Table 2.** Quantification of the Specific Putaminal Uptake at Baseline, 1 Month, and 1 Year After GD
- **Supplementary material, videos**

**Supplementary Table 1. Characteristics and Past Medical History of Patients**

|  | **Patient 1** | **Patient 2** |
| --- | --- | --- |
| Diagnosis of AADC Deficiency | | |
| Birth | Non-consanguineous parents; uneventful pregnancy; birth parameters: weight, 2.85 kg; length, 49.5 cm; cranial perimeter, 35 cm; Apgar score 9 | Consanguineous parents; uneventful pregnancy; birth parameters: weight, 3.26 kg; length, 51 cm; cranial perimeter, 36.5 cm; Apgar score, 10 |
| Age at onset of the symptoms | 3 mo | Neonate |
| Age at diagnosis | 3.8 y | 6 mo |
| *DDC* variant | Homozygous pathogenic variant c.1040G>A (p.Arg347Gln) | Homozygous pathogenic variant c.1040G>A (p.Arg347Gln) |
| AADC enzyme activity | No AADC activity detection in blood | No AADC activity detection in blood |
| Symptoms at diagnosis | Hypotonia with no head control  Feeding difficulties  Early onset irritability with frequent crying  Sleep disturbances  Diurnal fluctuation | Neonatal hypotonia  Feeding difficulties  Stridor |
| Overall symptom history | | |
| Motor function | Developmental delay  Hypokinesia  No head control  Limited voluntary movements  Distal dystonia | Developmental delay  Hypokinesia  No head control  Limited voluntary movements |
| Oculogyric crisis age at onset/at last follow-up | From 7 mo  Twice weekly for 4–6 h involving the eyes, face, neck | From 6 mo decreased after 5.5 y  Twice yearly |
| Mood/sleep | Sleep disturbances  Irritability  Excessive crying, partial improvement at 9 y  Good eye contact  Smiles | Sleep disturbances  Irritability  Excessive crying  Good eye contact  Smiles |
| Digestive function | Blended meals only  Constipation from 1–6 y | Gastrostomy at 6 y  3 stools/d after 8 y  Diarrhea |
| Respiratory function | No dysfunction or infections | Intermittent non-invasive ventilation until 10 y  Systematic antibiotic prophylaxis  Recurrent infection every 2 mo requiring antibiotherapy |
| Other findings | Intense nasal congestion until 3 y  Temperature instability until 3 y  Excessive hyperhidrosis until 3 y  Diurnal fluctuation of symptoms | Nasal congestion until 7 y  Brief episodes of temperature instability every 3 mo  Focal epileptic seizures from 6 y treated by sodium valproate and clonazepam  Diurnal fluctuation of symptoms |
| Previous treatments | Vitamin B_6_, pyridoxal phosphate, pramipexole, selegiline, 5-HTP, amitriptyline, folinic acid, melatonin, methylphenidate | Pramipexole, bromocriptine, trihexyphenidyl, l-DOPA |

5-HTP, 5-hydroxytryptophan; AADC, aromatic l-amino acid decarboxylase; *DDC*, dopa decarboxylase; l-DOPA, l-3,4-dihydroxyphenylalanine.

**Supplementary Table 2. Quantification of the Specific Putaminal Uptake at Baseline, 1 Month, and 1 Year After GD**

The SPU is defined by $SPU=\frac{{SUV}_{max}^{right and left putamen}-{SUV}_{max}^{occipital cortex}}{{SUV}_{max}^{occipital cortex}}$, where${SUV}_{max}$ is the maximal standardized uptake value in a region of interest. These measurements confirm the increase of the specific putaminal uptake at 1 month, an increase that persists at 1 year for Patient 1, but fragments and decreases after 1 month for Patient 2, without returning to baseline.

| SPU | Patient 1 | Patient 2 |
| --- | --- | --- |
| Baseline | 0.15 | 0.07 |
| 1 mo | 0.33 | 0.38 |
| 1 y | 0.36 | 0.24 |

GD, gene delivery; SPU, specific putaminal uptake; SUV, standard uptake value.
